# Clock Genes Regulate the Circadian Expression of *Piezo1*, *TRPV4*, *Connexin26*, and *VNUT* in an *Ex Vivo* Mouse Bladder Mucosa

**DOI:** 10.1371/journal.pone.0168234

**Published:** 2017-01-06

**Authors:** Tatsuya Ihara, Takahiko Mitsui, Yuki Nakamura, Satoru Kira, Hiroshi Nakagomi, Norifumi Sawada, Yuri Hirayama, Keisuke Shibata, Eiji Shigetomi, Yoichi Shinozaki, Mitsuharu Yoshiyama, Karl-Erik Andersson, Atsuhito Nakao, Masayuki Takeda, Schuichi Koizumi

**Affiliations:** 1 Department of Urology, Interdisciplinary Graduate School of Medicine, University of Yamanashi, Chuo, Yamanashi, Japan; 2 Department of Immunology, Interdisciplinary Graduate School of Medicine, University of Yamanashi, Chuo, Yamanashi, Japan; 3 Department of Neuropharmacology, Interdisciplinary Graduate School of Medicine, University of Yamanashi, Chuo, Yamanashi, Japan; 4 Wake Forest University, Institute for Regenerative Medicine, Winston-Salem, North Carolina, United State of America; Pennsylvania State University, UNITED STATES

## Abstract

**Objectives:**

*Clock*^*Δ19/Δ19*^ mice is an experimental model mouse for nocturia (NOC). Using the bladder mucosa obtained from *Clock*^*Δ19/Δ19*^ mice, we investigated the gene expression rhythms of mechanosensory cation channels such as transient receptor potential cation channel subfamily V member 4 (*TRPV4*) and *Piezo1*, and main ATP release pathways including vesicular nucleotide transporter (*VNUT)* and *Connexin26*(*Cx26*), in addition to clock genes.

**Materials and methods:**

Eight- to twelve-week-old male C57BL/6 mice (WT) and age- and sex-matched C57BL/6 *Clock*^*Δ19/Δ19*^ mice, which were bred under 12-h light/dark conditions for 2 weeks, were used. Gene expression rhythms and transcriptional regulation mechanisms in clock genes, mechanosensor, *Cx26* and *VNUT* were measured in the mouse bladder mucosa, collected every 4 hours from WT and *Clock*^*Δ19/Δ19*^ mice using quantitative RT-PCR, a Western blot analysis, and ChIP assays.

**Results:**

WT mice showed circadian rhythms in clock genes as well as mechanosensor, *Cx26* and *VNUT*. Their expression was low during the sleep phase. The results of ChIP assays showed Clock protein binding to the promotor regions and the transcriptional regulation of mechanosensor, *Cx26* and *VNUT*. In contrast, all of these circadian expressions were disrupted in *Clock*^*Δ19/Δ19*^ mice. The gene expression of mechanosensor, *Cx26* and *VNUT* was maintained at a higher level in spite of the sleep phase.

**Conclusions:**

Mechanosensor, *Cx26* and *VNUT* expressed with circadian rhythm in the mouse bladder mucosa. The disruption of circadian rhythms in these genes, induced by the abnormalities in clock genes, may be factors contributing to NOC because of hypersensitivity to bladder wall extension.

## Introduction

The products of clock genes act as transcription factors, are expressed in most cells and organs. The products of clock genes produce oscillations in sleep-awake rhythms and the gene expression of various metabolic enzymes, channels, and receptors with circadian rhythms. Among more than 10 types of clock genes that have been identified to date, Clock and Bmal1 protein dimers bind to E-box enhancer elements, which exist in the promoter sequence of target genes such as *Per*, *Cry*, and *Clock*, and clock-controlled genes, and then activate the transcription of these genes. The translational products of *Per* and *Cry* inhibit Clock and Bmal1 protein dimers. As a result, the transcription of these genes is down-regulated. These translational cycles are known as the ‘core loop’. Other feedback loops are known as ‘sub loops’, which are created by other clock genes: e.g. the products of *Dbp* and *E4bp4* bind to the D-box site while those of *RORα* and *Rev-erbα* bind to the RORE site. Exact circadian gene expression is driven by the formation of a large number of complex feedback loops under the control of the master clock in the suprachiasmatic nucleus (SCN) [1].

Nocturia (NOC) is exceedingly common general complain. It is defined as the waking up at night one or more times to void [2], which is reported that nearly 90% of the elderly men are suffering from NOC [3]. With the prevalence of NOC, it raise the various risk such as sleeping disorder, mental health, bone fracture by fall and reduce the life span [4–7]. Many diseases cause NOC, and the palliative treatments depending on the causes, such as α_1_-blockers for benign prostatic hyperplasia, anti-cholinergic drugs or β_3_-agonists for an overactive bladder, desmopressin for nocturnal polyuria, anti-hypertensive drugs and diuretic medications for high blood pressure patients, are provided in clinical settings [8]. However, because the pathophysiologies of NOC are multifactorial and remain unclear in a large number of patients, these treatments are often not so effective and become refractory. So far, there was no ideal animal in order to examine the complicated pathophysiology of NOC.

We previously reported that *Clock* mutant mice showed the phenotype of NOC [9]. Furthermore, other groups found that that urine products and lower urinary tract function are regulated by clock genes [10, 11]. These findings provided us with a concern regarding the relationship between abnormalities in clock genes and lower urinary tract symptoms (LUTS). On the other hand, the bladder mucosa senses bladder extension and transmits signals to afferent nerves by releasing neurotransmitters, including adenosine triphosphate (ATP) [12–15]. Bladder extension is sensed by mechanosensor such as transient receptor potential cation channel subfamily V member 4 (*TRPV4)* and *Piezo1* via intracellular Ca^2+^ influx [16–18], which triggers ATP release from the bladder urothelium. Although many pathways are involved in the release of ATP, we identified the vesicular nucleotide transporter (*VNUT)*, that mediates exocytosis [19] and conductive release connexin- or pannexin-hemichannels that mediate diffusible ATP release in the mucosa [20–25], particularly *Connexin26* (*Cx26*) [26], as a main ATP release pathway in the bladder mucosa.

Based on these findings, we hypothesize that the expression of mechanosensor such as *Piezo1* and *TRPV4*, and main ATP pathway such as *Cx26* and *VNUT*, is regulated by clock genes in the bladder mucosa, and these create a circadian rhythm for the sensation of bladder fullness. Moreover, abnormalities in clock genes enforce hypersensitive to mechanosensory stimuli upon the bladder mucosa during sleep because of a disruption in the circadian rhythms of mechanosensor, *Cx26* and *VNUT*.

In the present study, we investigated whether the gene expression of mechanosensor, *Cx26* and *VNUT* show circadian rhythms in the bladder mucosa, and also if they are regulated by clock genes.

## Materials and Methods

### Animals

Eight- to twelve-week-old male C57BL/6 mice (WT; SLC, Shizuoka, Japan) and age- and sex-matched C57BL/6 *Clock* mutant mice (*Clock*^*Δ19/Δ19*^; Jackson Laboratories, Bar Harbor, ME, USA) were used in the following experiments. For genotyping of *Clock*^*Δ19/Δ19*^ mice, polymerase chain reaction was performed using the following primers: WT forward, 5’GGTCAAGGGCTACAGGTA-3’; common, 5’TGGGGTAAAAAGACCTCTTGCC-3’; mutant forward, 5’AGCACCTTCCTTTGCAGTTCG-3’; mutant reverse, 5’TGTGCTCAGACAGAATAAGTA-3’. Mice were sacrificed by cervical dislocation after the anesthesia using sevoflurane to minimize the animal suffering. *Clock*^*Δ19/Δ19*^ mice have an A to T mutation in the 5’ splice site of intron 19, and, as a consequence, an in-frame deletion of the entire exon 19 (*Clock*^*Δ19/Δ19*^). The product of *Clock*^*Δ19/Δ19*^ results in the loss of normal transcriptional activity as a transcription factor [27].

All experiments were performed using these mice, which were bred under 12-h light/dark conditions for 2 weeks. The light period started from 6 AM, which is zeitgeber time (ZT) 0, and the dark period started from 6 PM, which is ZT 12.

All procedures were conducted in accordance with the Guiding Principles in the Care and Use of Animals in the Field of the Physiological Society of Japan, all experiment were approved by the Institutional Animal Care and Use Committee of the University of Yamanashi (Chuo, Yamanashi, Japan).

### Collection method for the mouse bladder mucosa

Mice were sacrificed every 4 hr from ZT0, and bladders were removed and everted. The inner surface (mucosa) of the bladder was scraped with a knife in PBS (S1 Fig). Suspensions including the mouse bladder mucosa were centrifuged at 1,000 rpm for 5 min, and cell pellets were collected. All samples were lysed in Buffer RLT (Qiagen, Germany) to extract mRNA for the quantitative real-time reverse transcription polymerase chain reaction (RT-PCR) and in M-PER Mammalian Protein Extraction Reagent (Thermo Fisher Scientific, USA) to extract protein for a Western Blotting analysis.

### Histological examination of mouse bladder tissues

Frozen tissues embedded in OCT compound (Sakura Finetek Japan, Tokyo, Japan) were cut into 7-μm-thick sections. Staining with standard hematoxylin and eosin (H&E) and Masson’s trichrome was performed in order to compare morphological changes before or after scraping of the bladder mucosa.

### Quantitative real-time RT-PCR

Total RNA was isolated and purified from the mouse bladder mucosa using an RNeasy mini kit (Qiagen, Germany) according to the manufacturer’s instructions. RNA was quantified using a spectrophotometer (GeneQuant, Biochrom, Cambridge, UK). Approximately 50 ng RNA was reverse transcribed using a first-strand cDNA synthesis kit (Roche, Basel, Switzerland). Reverse transcription was performed at 50°C for 60 min, followed by 85°C for 5 min to inactivate reverse transcriptase. Quantitative real-time RT-PCR was performed using SYBR^®^ premix Ex taq (Takara Bio, Inc., Japan) and Primer random p(dN)_6_ (Roche Diagnosis GmbH, Mannheim, Germany) in a Smart Cycler System (Cepheid, Sunnyvale, Calif). Gene-specific primers were designed using the online program Primer 3. Primer sequences are shown in Table 1.

**Table 1:** Primer sequences.

Gene symbol	Accession No	Primers for RT-PCR	sequence (5'→3')
m*Tbcc*	NM_178385	Fw	GACTCCTTCCTGAACCTCTGG
		Rv	GGAGGCCATTCAAAACTTCA
m*Eif2a*	NM_001005509	Fw	CAACGTGGCAGCCTTACA
		Rv	TTTCATGTCATAAAGTTGTAGGTTAGG
m*Per2*	NM_011066	Fw	TGGTTTCTGGGAAGATCCTG
		Rv	CCACAAACTTGGCATCACTG
m*Bmal1*	NM_007489	Fw	CGCCGCTCTTTCTTCTGTAG
		Rv	GGTGGCCAGCTTTTCAAATA
m*Cry1*	NM_007771	Fw	TCCCCTCCCCTTTCTCTTTA
		Rv	TTCTTGTCCCAAGGGATCTG
m*Clock*	NM_007715	Fw	GGTAACGCGAGAAAGATGGA
		Rv	AGCATCTGACTGTGCAGTGG
m*Dbp*	NM_0169745	Fw	GCCCACTTGGTACAGAAGGA
		Rv	CTGCAGAAAGGTGCAACTCA
m*E4bp4*	NM_017373	Fw	TTCTGATGGGGAAGACGAAC
		Rv	TTCACTTCCGGAACCTTCAC
m*RORα*	NM_013646	Fw	TCCTTCACCAACGGAGAGAC
		Rv	CCAGGTGGGATTTGGATATG
m*Rev-erbα*	NM_145434	Fw	CTGCAGGCTGATTCTTCACA
		Rv	TCTTGGGGTGGCTATACTGC
m*Piezo1*	NM_001037298	Fw	ATCCTGCTGTATGGGCTGAC
		Rv	AAGGGTAGCGTGTGTGTTCC
m*TRPV4*	NM_022017	Fw	TCACCTTCGTGCTCCTGTTG
		Rv	AGATGTGCTTGCTCTCCTTG
m*Connexin26*	NM_008125	Fw	ACTCCACCAGCATTGGAAAG
		Rv	ACAAAATCGGCTTGCTCATC
m*VNUT*	NM_183161	Fw	CAGAGTCATCACGGTGCGTAA
		Rv	GACCCAGACACAGGGCAAA

Reverse transcription products were subjected to 45 cycles of PCR, with a thermal program at 95°C for 5 sec for denaturation, and at 60°C for 20 sec for annealing, elongation, and detection. mRNA levels were calculated from the standard curve, which ran simultaneously with sample tubes, and was normalized as a ratio to *Eif2a/Tbcc* concentrations [12]. PCR products were electrophoresed on a 2.5% agarose gel to confirm the target band size.

### Western blotting analysis

The protein concentration of each lysed sample by M-PER Mammalian Protein Extraction Reagent (Thermo Fisher Scientific, USA) was measured using Pierce 660nM Protein Assay Reagent (Thermo Fisher Scientific, USA). Samples were diluted to the reference concentration, which was the lowest concentrated sample. Diluted lysates were then subjected to sodium dodecyl sulfate-polyacrylamide gel electrophoresis (SDS-PAGE) on 7.5% gels using a Power station 1000VC system (Atto, USA) at 20 mA for 90 min. Proteins were transferred to polyvinylidene fluoride (PVDF) membranes using a Power Pac (Bio-Rad, USA) at 70 V for 120 min. The transferred membrane was cut into several strips according to the product size of target protein. The stripped membranes were blocked with 2% ECL prime Blocking Agent (GE Life Sciences, Japan) at room temperature (RT) for 1 h and then washed 3 times with PBS independently. The each membrane was incubated with the following first antibodies diluted with Can Get Signal^®^ solution 1 (Toyobo, Japan) at 4°C overnight: a rabbit anti-β-Actin antibody (Santa Cruz; 1:5000), rabbit anti-Clock antibody (Cell Signaling Technology; 1: 800), rabbit anti-Bmal1 antibody (Abcam; 1:800), rabbit anti-Piezo1 antibody (Novus; 1:500), rabbit anti-TRPV4 antibody (Santa Cruz; 1:800), rabbit anti-Connexin26 antibody (Invitrogen; 1:100), and rabbit anti-VNUT antibody (Millipore; 1:500). After washing 3 times, membranes were incubated with horseradish peroxidase-conjugated anti-rabbit antibodies (1:6000; Amersham Pharmacia Biotech Inc., Piscataway, NJ, USA) diluted with Can Get Signal^®^ solution 2 (Toyobo, Japan) at RT for 1 h. The proteins were visualized as bands by chemiluminescence ECL select Western Blotting Detection Reagent (GE Life Sciences, Japan).

### Chromatin immunoprecipitation assay (ChIP)

The ability of Clock to bind to the promoter regions of *Piezo1*, *TRPV4*, *Cx26*, and *VNUT* in the mouse bladder mucosa was analyzed at different time points using ChIP assays. ChIP assays were performed using a Simple ChIP^®^ plus enzymatic chromatin IP kit (Cell Signaling technology, USA) according to the manufacturer’s instructions. The mouse bladder mucosa was obtained at ZT0 and ZT8 or ZT4 and ZT12 depending on the peak and nadir time in each gene. Samples were cross-linked with formaldehyde solution (Sigma-Aldrich, USA) and lysed, and chromatin was fragmented by partial digestion with Micrococcal Nuclease in order to obtain chromatin fragments of 1 to 5 nucleosomes. ChIP assays were performed with ChIP-Grade Protein G Agarose Beads, using a negative control rabbit IgG and rabbit anti-Clock antibody (Cell Signaling Technology; 1: 100). After the reversal of protein-DNA cross-links, DNA was purified. The amounts of Clock bound to target DNA were quantified in 50 cycles of real-time PCR using primers and Taq Man probes using Taq Man^®^ Fast Advanced Master Mix (Applied Biosystems, USA), with a thermal program of 95°C for 15 sec for denaturation and 60°C for 60 sec for annealing, elongation, and detection in a Smart Cycler System (Cepheid, Sunnyvale Calif). The ratio of a specific DNA fragment in each immunoprecipitated sample (IP sample) to that fragment in DNA before immunoprecipitation (input DNA) was calculated from each cycling threshold cycle of PCR reaction (C[T]) using the equation described in the manufacturer’s instructions as *Percent Input = 2%×2*^*C[T] input DNA–C[T] IP sample*^. The primers and Taq Man probes used were listed in Table 2.

**Table 2:** Primer and Taq Man probe sequences.

Gene symbol	Primers for ChIP assays	sequence (5'→3')
m*Per2* E2	Sense	CCACCAATTGATGAGCGTAGC
	Antisense	CGTCGCCCTCCGCTG
	Taq Man probe	FAM-TCACGTTTTCCACTATGTG-MGB
m*Per2* E5	Sense	TCCTGCCACATTGAGATTTGG
	Antisense	GTGATTGCCCCACACTCACA
	Taq Man probe	FAM-AAGAGATGGCACGTTAGT-MGB
m*Piezo1*	Sense	GCTCATGGCCTTTTAACCTTACCT
	Antisense	CACCTCGGGCAATTGCTATTTT
	Taq Man probe	FAM-TAGGACAGTCAGTTTCTG-MGB
m*TRPV4*	Sense	CCTAGGAAAAGGAACGGTGATGTTA
	Antisense	GAACCTCCACCTATAGCCATCATC
	Taq Man probe	FAM-CCCCTAGCATCTTCC-MGB
m*Connexin26*	Sense	AAGTGACCTCAGCCAAGAAACTAC
	Antisense	GGCTGCCCAGCTCGTAT
	Taq Man probe	FAM-CAGAATTTGTGAAATCCC-MGB
m*VNUT*	Sense	GGGTAGAACCAACGGAGACTCATA
	Antisense	CGAGACCCGCCAGAAATAACTTT
	Taq Man probe	FAM-CACACTGCTCACCTGCTC-MGB

### Statistical analyses

Experimental values were expressed as means ± standard error (SE). The significance of differences between two groups was analyzed using Mann-Whitney’s *U*-test. A one-way ANOVA was used to compare differences among the time points in each group. A two-way ANOVA with Bonferroni’s test was used to compare differences at each time point between 2 groups. A *P* value of less than 0.05 was considered significant.

## Results

### 1. Circadian experiments on clock genes

**Fig 1:**
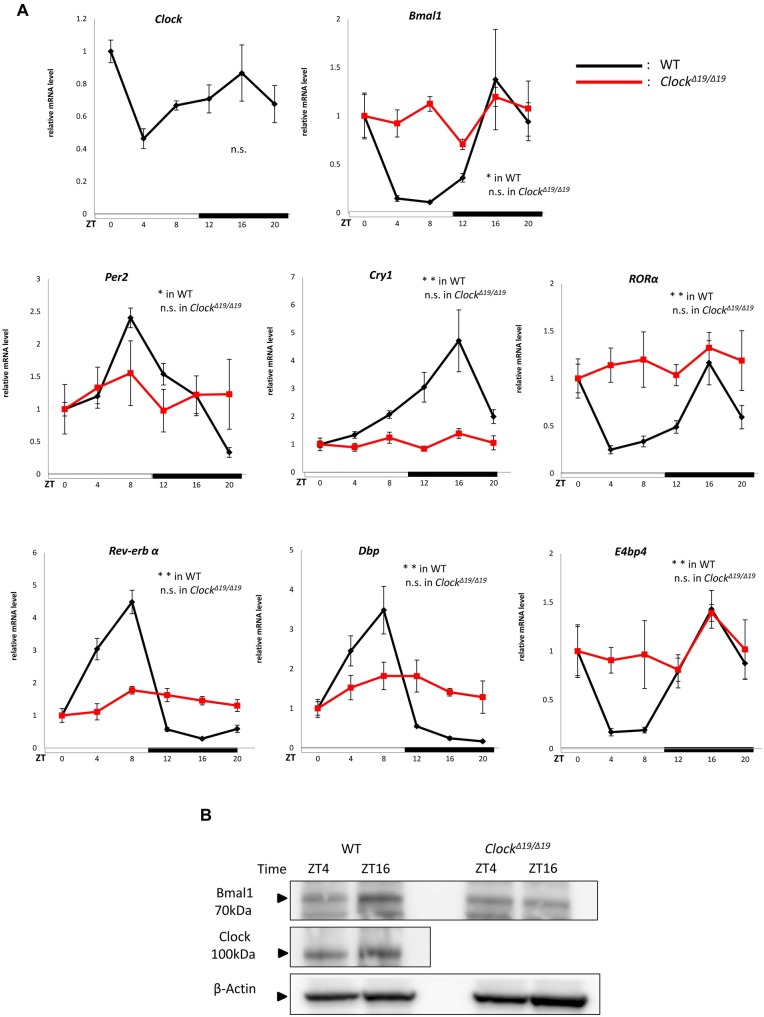
Time-dependent changes in clock gene expression in the mouse bladder mucosa. (A) Clock gene mRNA expression rhythms in the mouse bladder mucosa in WT and *Clock*^*Δ19/Δ19*^ mice under a 12-h light/dark cycle. (B)Representative band image of *Clock* and *Bmal1* proteins between ZT4 and ZT16 in WT and *Clock*^*Δ19/Δ19*^ mice under a 12-h light/dark cycle. N = 4 for WT mice, N = 4 for *Clock*^*Δ19/Δ19*^ mice at each point. Statistical analyses were performed using a one-way ANOVA to compare differences among time points in each group. *P < 0.05, **P < 0.01, n.s., not significant. Data are presented as means ± SE. ZT0 time point was normalized as 1 independently in each genotype.

We confirmed the circadian expression of clock gene mRNA using quantitative RT-PCR in the mouse bladder mucosa in WT and *Clock*^*Δ19/Δ19*^ mice (n = 4 for WT mice, n = 4 for *Clock*^*Δ19/Δ19*^ mice at each time point, with a total of 24 mice in each group) (Fig 1A). The clock genes examined were *Per2*, *Bmal1*, *Cry1*, and *Clock* as core loop clock genes, and *Dbp*, *E4bp4*, *RORα*, and *Rev-erbα* as sub-loop clock genes [1].

In WT mice, *Clock* mRNA appeared to have a circadian rhythm with a peak at ZT0 and nadir at ZT4. *Per2* mRNA showed a circadian rhythm with a peak at ZT8 and nadir at ZT20. In contrast, *Bmal1* mRNA showed a circadian rhythm with a peak at ZT16 and nadir at ZT8. The expression rhythms between *Per2* and *Bmal1* were inversed; *Per2* mRNA levels as a negative transcription factor were low during the active phase and high during the sleep phase, while *Bmal1* mRNA levels as a positive transcription factor were high during the active phase and low during the sleep phase. The rhythm of *Cry1* was similar to that of *Bmal1* because *Cry1* functions as a suppressor of *Bmal1* function. *RORα* as a positive transcription factor and *Rev-erbα* as a negative transcription factor, which creates circadian expression through the RORE site, showed the same circadian patterns as *Per2* and *Bmal1*, respectively. *Dbp* as a positive transcription factor and *E4bp4* as a negative transcription factor, which create circadian expression through D-box, also showed the same circadian patterns as *Per2* and *Bmal1*, respectively. In contrast, the circadian mRNA expressions of these clock genes were disrupted in *Clock*^*Δ19/Δ19*^ mice (Fig 1A).

In the Western blotting analysis, Bmal1 protein expression showed a diurnal change between ZT4 and ZT16 in WT mice (Fig 1B), which was consistent with the peak and nadir of *Bmal1* mRNA expression. In the case of the Clock protein, a change in band density was not observed between ZT4 and ZT16 (Fig 1B), which was consistent with *Clock* mRNA expression. In contrast, *Clock*^*Δ19/Δ19*^ mice showed the loss of a diurnal change in the Bmal1 protein, which was consistent with *Bmal1* mRNA expression in *Clock*^*Δ19/Δ19*^ mice (Fig 1B).

### 2. Circadian experiments on mechanosensor, *Cx26* and *VNUT*

Subsequent to the circadian rhythms of clock genes, the circadian mRNA expression of mechanosensor such as *Piezo1* and *TRPV4*, and main ATP pathway including *Cx26* and *VNUT* was examined in the mouse bladder mucosa (n = 4 for WT mice, n = 4 for *Clock*^*Δ19/Δ19*^ mice at each time point, with a total of 24 mice in each group).

**Fig 2:**
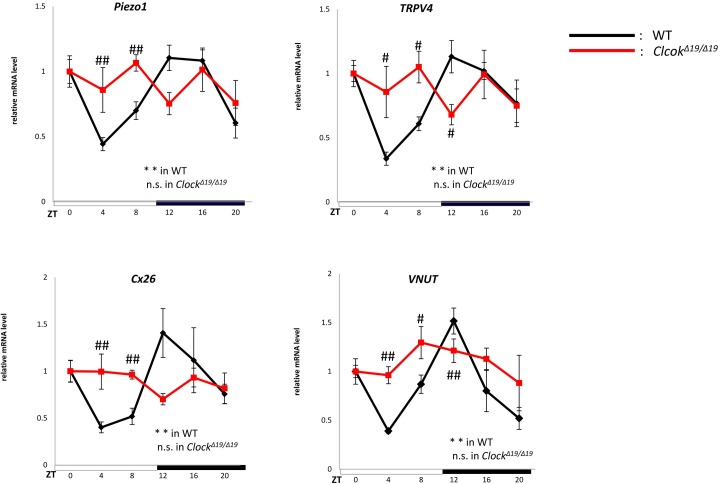
Time-dependent changes in mechanosensor,*Cx26* and *VNUT* mRNA expression in the mouse bladder mucosa. Mechanosensor,*Cx26* and *VNUT* mRNA expression rhythms in the mouse bladder mucosa in WT and *Clock*^*Δ19/Δ19*^ mice under a 12-h light/dark cycle. N = 4 for WT mice, N = 4 for *Clock*^*Δ19/Δ19*^ mice at each point. Statistical analyses were performed using a one-way ANOVA to compare differences among time points in each group. *P < 0.05, **P < 0.01, n.s., not significant. Data are presented as means ± SE. ZT0 was normalized as 1 independently in each genotype. Using S2 Fig, a two-way ANOVA and Bonferroni’s test was used to compare differences of absolute mRNA level between WT and *Clock*^*Δ19/Δ19*^ mice at each time point (S2 Fig). # *P* < 0.05, ## *P* < 0.01.

In WT mice, mRNA in *Piezo1*, *TRPV4*, *Cx26*, and *VNUT* showed circadian changes in expression (Fig 2). Peaks were observed at ZT12 at the beginning of the active phase, and nadirs were at ZT4 at the middle of the sleep phase. The peaks and nadirs of these genes were slightly earlier than those of *Per2* and *Bmal1* in Fig 1A. In contrast, the mRNA expression of *Piezo1*, *TRPV4*, *Cx26*, and *VNUT* lost circadian rhythms and was observed at the same level between ZT0 and ZT20 in *Clock*^*Δ19/Δ19*^ mice (Fig 2). The absolute mRNA levels of these genes were compared using unnormalized data (S2 Fig). The mRNA abundances between WT and *Clock*^*Δ19/Δ19*^ mice during the sleep phase (ZT4 and ZT8) were significantly higher in *Clock*^*Δ19/Δ19*^ mice than in WT mice. In contrast, the absolute mRNA levels of these genes during the active phase (ZT12) increased in WT mice. Significant differences were observed in *TRPV4* and *VNUT* between WT mice and *Clock*^*Δ19/Δ19*^ mice.

**Fig 3:**
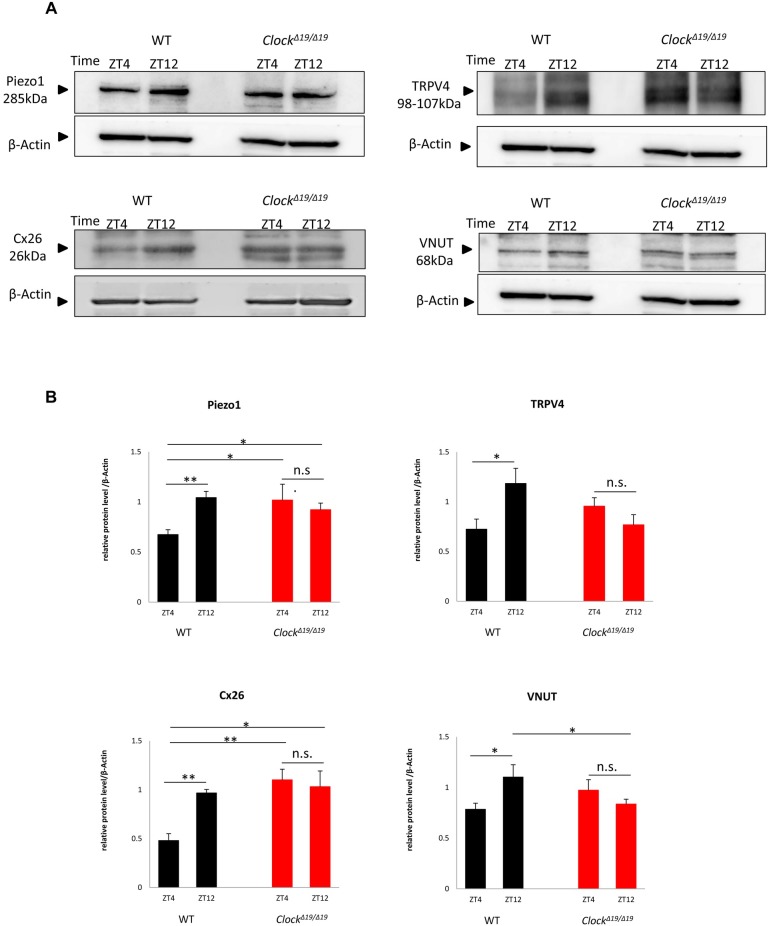
Protein expression rhythms of mechanosensor, *Cx26* and *VNUT* in the mouse bladder mucosa. (A) Representative band image of Piezo1, TRPV4, Cx26, and VNUT proteins by Western blotting of the mouse bladder mucosa of WT and *Clock*^*Δ19/Δ19*^ mice between ZT4 and ZT12. (B) Quantitative analysis of Piezo1, TRPV4, Cx26, and VNUT proteins by Western blotting of the mouse bladder mucosa of WT and *Clock*^*Δ19/Δ19*^ mice between ZT4 and ZT12. N = 5 for WT mice, N = 5 for *Clock*^*Δ19/Δ19*^ mice at each point in a protein quantitative analysis. *P < 0.05, **P < 0.01, n.s., not significant by Mann-Whitney’s *U*-test.

We then investigated diurnal changes in the protein expression of *Piezo1*, *TRPV4*, *Cx26*, and *VNUT*. The times for collecting samples were selected based on the nadir and peak points of mRNA expression (ZT4 as a nadir point and ZT12 as a peak point). In WT mice, the band densities of Piezo1, TRPV4, Cx26, and VNUT were lower at ZT4 than at ZT12 (Fig 3A). Quantitative analyses revealed that the protein abundance of Piezo1, TRPV4, Cx26, and VNUT were significantly greater at ZT12 than at ZT4 (Fig 3B) (n = 5 for WT mice at each time point). These diurnal changes were consistent with the circadian rhythms in the mRNA levels of mechanosensor, *Cx26* and *VNUT* (Fig 2).

In contrast, Piezo1, TRPV4, Cx26, and VNUT proteins in *Cloc*k^*Δ19/Δ19*^ mice were continuously expressed at the same level between ZT4 and ZT12 (Fig 3A). Quantitative analyses also showed the disruption of diurnal expression rhythms between ZT4 and ZT12 (Fig 3B), which was consistent with the circadian rhythms in mechanosensor, *Cx26* and *VNUT* mRNA expression in *Cloc*k^*Δ19/Δ19*^ mice (n = 5 for *Clock*^*Δ19/Δ19*^ mice at each time point) (Fig 2).

### 3. ChIP assays

In order to directly demonstrate that clock genes regulate the transcription of *Piezo1*, *TRPV4*, *Cx26*, *and VNUT* in the mouse bladder mucosa, we performed ChIP assays using an anti-Clock antibody. The integrity of ChIP samples was confirmed based on the constitutive binding of Clock to the promoter of the *Per2* gene containing the non-canonical E-box enhancer 2 (E2) sequence, but not the enhancer 5 (E5) sequence, as previously reported [27]. In the E2 region, significant Clock binding was observed at ZT8, but not at ZT0, which was consistent with the expression rhythms of *Per2* mRNA. In contrast, no Clock binding was noted in the E5 region at either time point (n = 3 for the Control group and anti-Clock Ab group at each time point) (S3 Fig).

**Fig 4:**
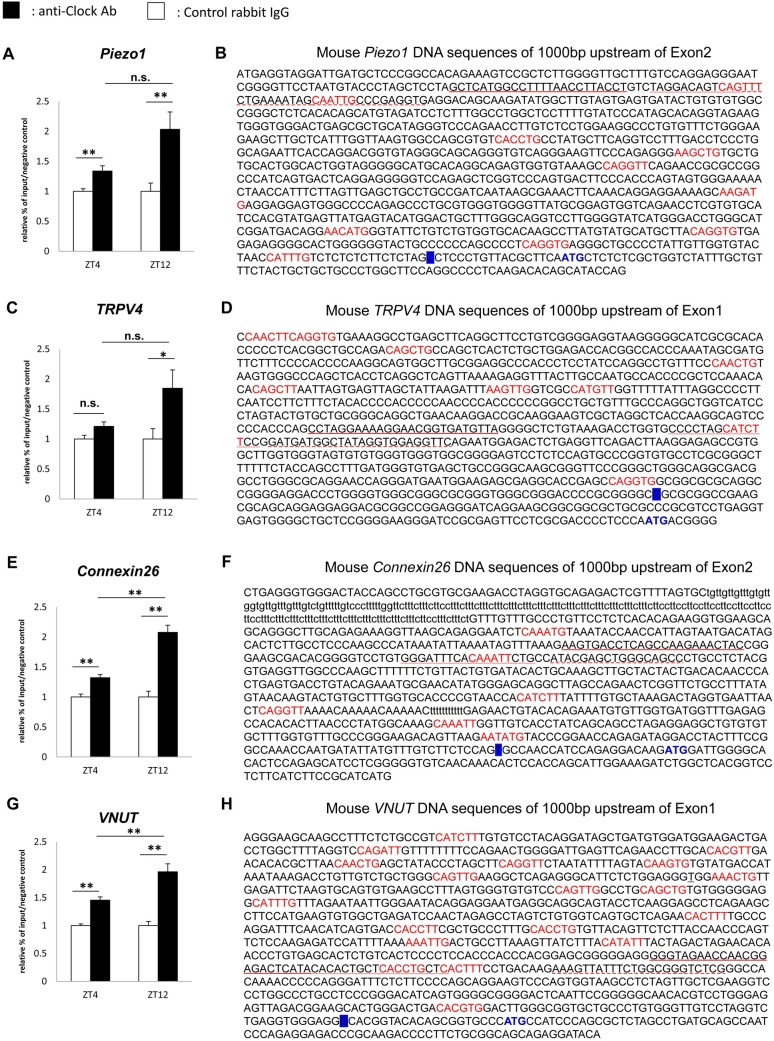
Detection of Clock binding to promoter regions. Clock binding to the E-box site on their sequences at ZT12 and ZT4, and these DNA sequences containing promoter regions and several non-canonical E-box-like elements. (A) and (B) *Piezo1*. (C) and (D) *TRPV4*. (E) and (F) *Cx26*. (G) and (H) *VNUT*. ‘‘CANNTG” or ‘‘CANNTT” or their reverse sequences are indicated in red. The transcription start site is labeled in blue. The start codon is indicated in blue. The underlined part indicates primers and probe sequence sites for the ChIP assay. The straight line indicates the sense primer, the broken line the antisense primer, and the wave line the Taq Man probe. The ChIP sample was obtained in 3 experiments. PCR was performed 3 times for each gene. Data are presented as a relative value to that of the input at each time point, and as means ± SE. (n = 9 for the Control group and anti-Clock Ab group at each time point). *P < 0.05, **P < 0.01, n.s., not significant by Mann-Whitney’s *U*-test. Each graph was described with the normalized value by the input DNA in each time point. The P values of the precipitated chromatin fragments using an anti-Clock antibody between ZT4 and ZT12 were 0.070 in *Piezo1* and 0.102 in *TRPV4*.

Several E-box-like elements, to which the Clock/Bmal1 complex may theoretically bind, are present in the promoter regions of *Piezo1*, *TRPV4*, *Cx26*, and *VNUT* (Fig 4B, 4D, 4F and 4H) [27, 28]. The amount of precipitated chromatin fragments obtained using the anti-Clock antibody was significantly higher at ZT4 and ZT12 in *Piezo1*, *Cx26*, *and VNUT* than that using control IgG. As for *TRPV4*, this significant difference was only observed at ZT12 (n = 9 for the Control group and anti-Clock Ab group at each time point), (Fig 4A, 4C, 4E and 4G). A comparison of differences in precipitated chromatin fragments using the anti-Clock antibody between ZT4 and ZT12 revealed significant changes in *Cx26* and *VNUT* (p = 0.0007 and 0.0031, respectively, by Mann-Whitney’s *U*-test). Furthermore, *Piezo1* and *TRPV4* showed slightly more Clock binding at ZT12 than at ZT4 (p = 0.070 and 0.102 by Mann-Whitney’s *U*-test). These differences in the sensitivities of Clock binding to the E-box-like element appear to be consistent with the peaks and nadirs in the mRNA of *Piezo1*, *TRPV4*, *Cx26*, and *VNUT* (Fig 2B).

## Discussion

We herein demonstrated that clock genes exist and create typical circadian expression profiles in the bladder mucosa derived from WT mice. In addition, the expression rhythms of mechanosensor (*Piezo1* and *TRPV4*), *Cx26* and *VNUT*, which are involved in the sensation of bladder fullness, also showed circadian rhythms associated with the circadian expression of clock genes. These products in mechanosensor, *Cx26* and *VNUT* increased during the active phase and decreased during the sleep phase in WT mice. ChIP assays showed the transcriptional regulation of these genes by Clock binding at their promoter sequences. On the other hand, *Clock*^*Δ19/Δ19*^ mice, a model mouse for NOC [9], showed disrupted circadian expression not only in clock genes, but also in mechanosensor, *Cx26* and *VNUT*. These results suggest that the sensation of bladder fullness may change in a time-dependent manner in WT mice. In contrast, *Clock*^*Δ19/Δ19*^ mice may constantly sense bladder fullness between the sleep and active phases due to the loss of the circadian expression of mechanosensor, *Cx26* and *VNUT*.

Treatments for NOC are often ineffective clinically, and unknown causes have been suggested to influence the incidence of NOC. We previously demonstrated that *Clock*^*Δ19/Δ19*^ mice showed the NOC phenotype, and abnormalities in clock genes may be one of the causes of NOC [9]. In order to investigate etiologies regarding a disruption in the circadian rhythm of voiding behavior in *Clock*^*Δ19/Δ19*^ mice, we focused on the gene expression rhythms of clock genes, mechanosensor, *Cx26* and *VNUT* in the mouse bladder mucosa, in which mechanosensor, *Cx26* and *VNUT* were reported to sense bladder wall extension and transmit signals of bladder fullness to the CNS [16–18, 20–26].

In WT mice, core loop and sub loop clock genes showed typical mRNA expression patterns in the mouse bladder mucosa, as reported previously (Fig 1) [1]. The circadian expression of all clock genes was abrogated in *Clock*^*Δ19/Δ19*^ mice. These results demonstrated that clock genes exist in the mouse bladder mucosa and regulate exact circadian gene expression in WT mice. In addition to clock genes, mechanosensor, *Cx26* and *VNUT* mRNA are also expressed based on circadian rhythms in WT mice. The peaks observed in the mRNA expression of mechanosensor, *Cx26* and *VNUT* were consistent with the active phase, whereas the nadirs were consistent with the sleep phase (Fig 2). In contrast, the circadian expression of all clock genes, mechanosensor, *Cx26* and *VNUT* observed in WT mice was disrupted in *Clock*^*Δ19/Δ19*^ mice (Figs 1 and 2). Furthermore, the absolute mRNA of mechanosensor, *Cx26* and *VNUT* in the mouse bladder mucosa of *Clock*^*Δ19/Δ19*^ mice were maintained at significantly higher levels than those in WT mice during the sleep phase (Fig 2 and S2 Fig). Taken all these results together, *Clock*^*Δ19/Δ19*^ mice sense the sensation of bladder fullness more than WT mice during the sleep phase, resulting in the NOC phenotype in *Clock*^*Δ19/Δ19*^ mice [9].

We performed a Western blot analysis to confirm the relationship between circadian mRNA expression levels and the abundance of these products. Protein expression of clock genes, mechanosensor, *Cx26* and *VNUT* was detected between 2 time points: ZT4 and ZT16, the nadir and peak times for the mRNA expression of clock gene proteins, and ZT4 and ZT12 for that of mechanosensor, *Cx26* and *VNUT*.

In clock genes, *Bmal1* protein expression was associated with its mRNA expression. Protein levels were higher at ZT16 than at ZT4 in WT mice, but were almost equal between ZT4 and ZT16 in *Clock*^*Δ19/Δ19*^ mice (Fig 1B). The protein abundance of mechanosensor, *Cx26* and *VNUT* was significantly higher at ZT12 than at ZT4 in WT mice. In contrast, *Clock*^*Δ19/Δ19*^ mice lost these differences between ZT4 and ZT12, namely, protein abundance was maintained at a constant level between the active and sleep phases (Fig 3). Differences in time-dependent protein expression changes in clock genes, mechanosensor, *Cx26* and *VNUT* correlated with circadian mRNA expression in WT and *Clock*^*Δ19/Δ19*^ mice (Figs 1–3).

These results indicate that proteins also show circadian expression according to the circadian mRNA expression of mechanosensor, *Cx26* and *VNUT*. Moreover, these results indicate that the sensation of bladder fullness may be stronger in *Clock*^*Δ19/Δ19*^ mice than in WT mice due to the higher expression levels of mechanosensor, *Cx26* and *VNUT* in the sleep phase because of the lack of a negative transcription feedback loop.

Although only two time points of comparisons in protein expression may be insufficient and hard to assert enough discussion, we focused on the timing of the expression in clock genes between mRNA and protein. The timing of the expression of clock proteins in other organs was approximately 6 hrs later than that of mRNA expression in peripheral tissues such as the mouse liver [29]. In contrast, mRNA and protein rhythms were almost simultaneous in the CNS [30]. A number of mechanisms responsible for circadian gene regulation have been reported such as the indirect function of clock proteins as co-factors [10], RNA methylation cycles [31], protein anti-oxidant cycles [32], and protein ubiquitination cycles [33]. The interventions of these transcription factors, which make translational pathway to be complicated, may delay the timing of protein expression after transcription. In this view, the dynamics of gene expression include various processes, which differ in each cell and gene [34–36]. Specific genes endowed with important functions appear to be translated immediately after mRNA transcription [37]. The timing of protein expression after the transcription of mechanosensor, *Cx26* and *VNUT* mRNAs also seemed to be simultaneous in the bladder mucosa. In addition, receptors in the bladder, the functions of which change with circadian rhythms, were previously reported to act as regulators of circadian rhythms in the local area [38]. Possibly, circadian gene expression processes of mechanosensor, *Cx26* and *VNUT* under the regulation of clock genes may dominate substantial role to create circadian function of bladder, although another factors that contribute to maintain circadian rhythm could exists in the bladder.

In order to elucidate the molecular mechanisms underlying the circadian expression of mechanosensor, *Cx26* and *VNUT* in WT mice and their abrogation in *Clock*^*Δ19/Δ19*^ mice, we performed ChIP assays using an anti-Clock antibody on the mouse bladder mucosa. The results obtained demonstrated that the circadian expression of mechanosensor, *Cx26* and *VNUT* were regulated by Clock binding to the promoter region in the mouse mucosa (Fig 4), and were consistent with circadian mRNA expression rhythms in *Piezo1*, *TRPV4*, *VNUT*, *and Cx26* (Fig 2).

The sensitivity of mPer2 E2 to Clock binding, which was detected by ChIP, was weaker at ZT0 than at ZT12 (S3 Fig). Furthermore, this sensitivity was significantly stronger at ZT12 than at ZT4 for *Cx26* and *VNUT* (Fig 4E and 4G). *Piezo1* and *TRPV4* also showed slightly stronger Clock binding at ZT12 than at ZT4 (Fig 4A and 4C). The differences observed in sensitivity to Clock binding may mediate one of the reasons for differences in the timing of the peaks and nadirs among each gene.

The pathophysiology of NOC is multifactorial and complex and its etiology currently remains unclear in a large number of elderly patients. Based on the results of the present study using *Clock*^*Δ19/Δ19*^ mice, we advocate a new concept that abnormalities in clock genes may be one of the causes of NOC based on hypersensitivity to the sensation of bladder fullness during the sleep phase.

For the limitations in the present study, we used the bladder mucosa, which is including not only the epithelial cell layer but also the cells constituting lamina propria. These components except for bladder urothelium may affect the differences of the result: in the timing of the peak and nadir of the gene expression, in the timing of protein expression after transcription, and the sensitivity of Clock bindings at different time points. These phenomena may limit our discussion of the underlying mechanisms of the sensation of bladder fullness, the function of bladder urothelium involved in NOC, based on the results of the present study. In order to examine the relationship between molecular expression rhythms and functional circadian rhythms in mechanosensor, *Cx26* and *VNUT*, further studies are needed to investigate whether the functions of only the bladder urothelium, which senses urine storage, show the circadian rhythm according to the circadian expressions in mechanosensor, *Cx26* and *VNUT* under the conditions excluding the effects from another components cells of the bladder and CNS.

Furthermore, the sensitivity of Clock protein binding on the promoter regions of *Cx26* or *VNUT* was different between dark and light phase in ChIP experiment. However, the expression in Clock protein abundance did not seem to correlated to diurnal change. It seemed to be difficult to discuss about the mechanism of the differences of Clock protein binding in ChIP experiment. Although several possibilities could be raised such as behaviors of Bmal1 protein, DNA accessibility, or the differences of Clock protein abundances [39, 40], we must await further extensive studies to clarify these such as ChIP experiment under *Bmal1* knockdown condition, and Western blot experiments with finely separated time courses using *Clock*^*Δ19/Δ19*^ mice etc.

## Conclusions

We obtained three novel insights into NOC based on the results of the present study. The expression of mechanosensor, *Cx26* and *VNUT* is regulated by clock genes in the bladder mucosa. The sensation of bladder fullness may have a circadian rhythm due to the expression rhythms of mechanosensor, *Cx26* and *VNUT* in the bladder mucosa, which were sensitive during the active phase and insensitive during the sleep phase. The disruption of circadian rhythms in these channels may be one of the factors contributing to NOC. Our results provide a novel aspect of NOC as well as a deeper understanding of and new therapeutic concepts for NOC.

## Supporting Information

S1 Fig Scraping of the mouse bladder mucosa from the whole bladder. (A) Hematoxylin-Eosin stain (H-E) before scraping. (B) Masson-Trichrome stain (M-T) before scraping. (C) H-E after scraping. (D) M-T after scraping. Only the mouse bladder mucosa was removed from the lamina propria. L: lumen, the arrowhead indicates the bladder mucosa. Left panel: ×40 magnification, middle panel: ×100 magnification, Right panel: ×400 magnification.
(TIF)

S2 Fig Comparisons of mRNA abundance in the gene expression rhythm of mechanosensor, *Cx26* and *VNUT* in the mouse bladder mucosa. The absolute mRNA level of mechanosensor,*Cx26* and *VNUT* in the mouse bladder mucosa in WT and *Clock*^*Δ19/Δ19*^ mice under a 12-h light/dark cycle. N = 4 for WT mice, N = 4 for *Clock*^*Δ19/Δ19*^ mice at each point. Statistical analyses were performed using a two-way ANOVA and Bonferroni’s test in order to compare differences of absolute mRNA level between WT and *Clock*^*Δ19/Δ19*^ mice at each time point. # *P* < 0.05, ## *P* < 0.01.
(TIF)

S3 Fig Detection of Clock binding to the promoter region of mPer2. Each graph was described with the normalized value by the input DNA in each time point. Data are presented as a relative value of that of the control at each time point, and as means ± SE. (n = 3 for the Control group and anti-Clock Ab group at each time point). *P < 0.05, Mann-Whitney’s *U*-test, n.s., not significant.
(TIF)

## Abbreviations

ATP: adenosine triphosphate
ChIP: chromatin immunoprecipitation
*Clock* mutant: Clock^Δ19/Δ19^
CNS: central nervous system
Cx26: *Connexin**26*
LUTS: lower urinary tract symptoms
NOC: nocturia
RT-PCR: reverse transcriptase polymerase chain reaction
SE: standard error
SCN: suprachiasmatic nucleus
TRPV4: transient receptor potential cation channel subfamily V member 4
VNUT: vesicular nucleotide transporter
ZT: zeitgeber time
