# Supplementary figures and images for: Clock Genes Regulate the Circadian Expression of Piezo1, TRPV4, Connexin26, and VNUT in an Ex Vivo Mouse Bladder Mucosa
Source: PLoS One. 2017 Jan 6;12(1):e0168234. doi: 10.1371/journal.pone.0168234 (PMC5218463; doi:10.1371/journal.pone.0168234)

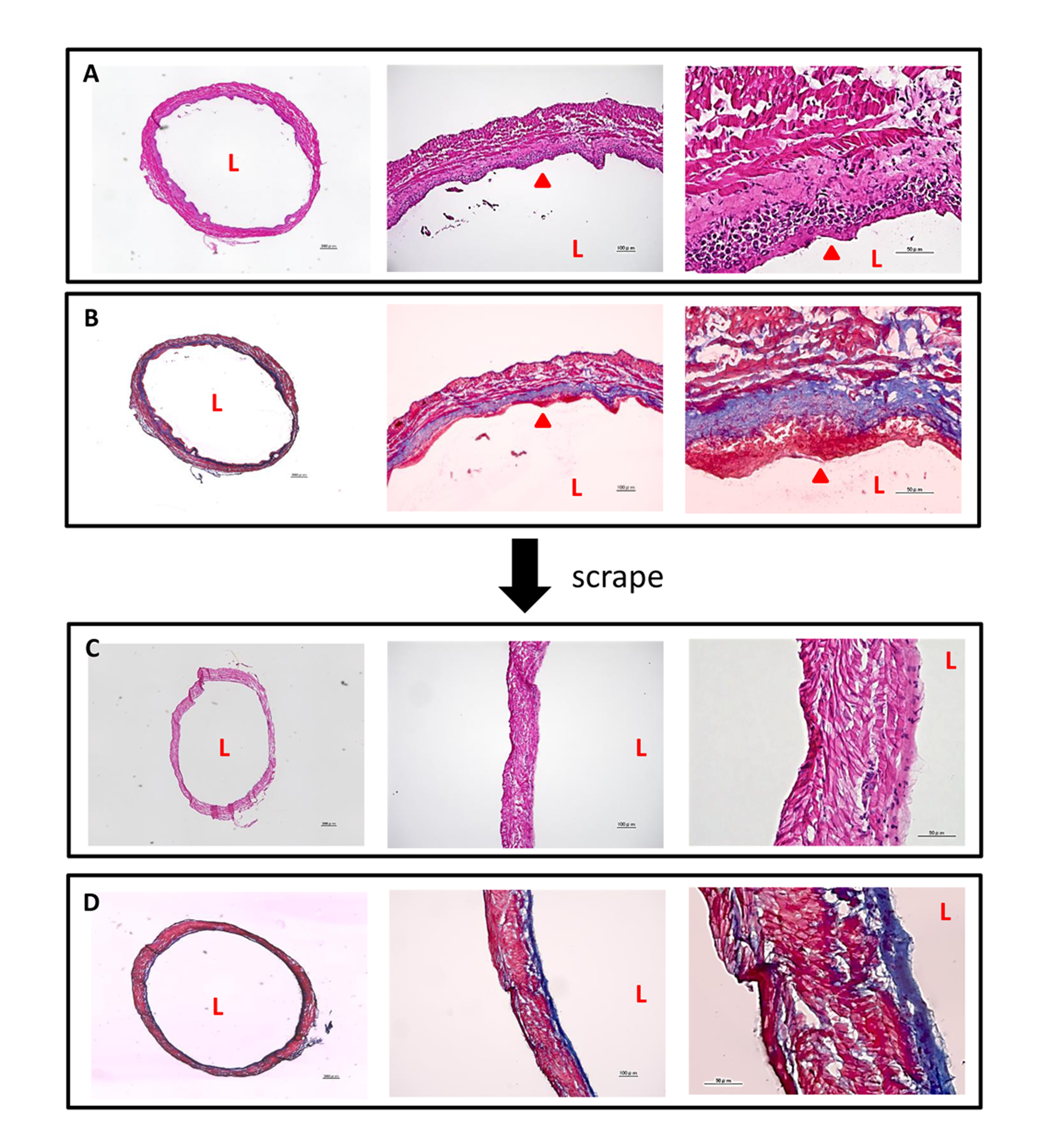

Supplement: S1 Fig — (A) Hematoxylin-Eosin stain (H-E) before scraping. (B) Masson-Trichrome stain (M-T) before scraping. (C) H-E after scraping. (D) M-T after scraping. Only the mouse bladder mucosa was removed from the lamina propria. L: lumen, the arrowhead indicates the bladder mucosa. Left panel: ×40 magnification, middle panel: ×100 magnification, Right panel: ×400 magnification. (TIF) [file pone.0168234.s001.tif]

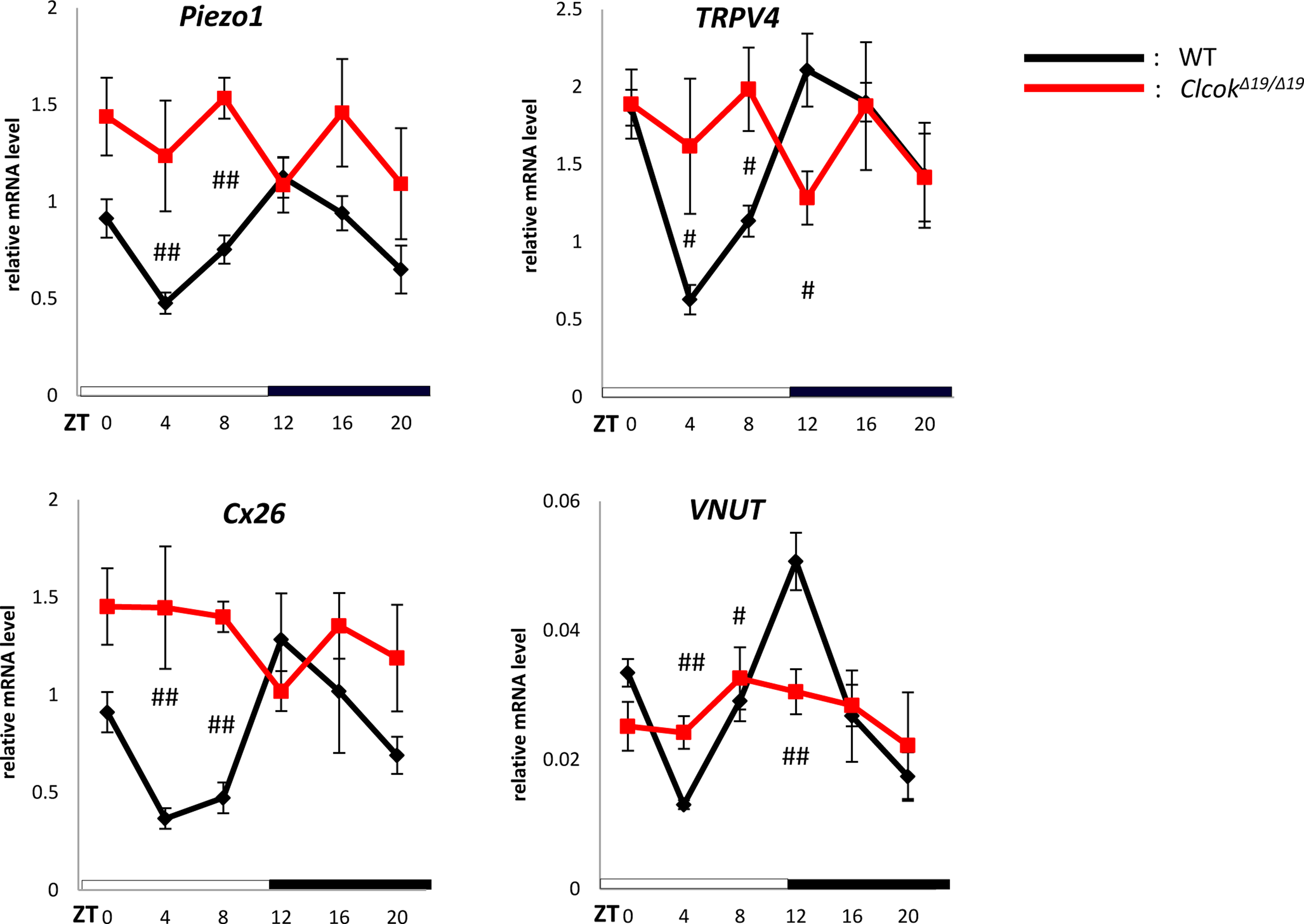

Supplement: S2 Fig — The absolute mRNA level of mechanosensor,Cx26 and VNUT in the mouse bladder mucosa in WT and ClockΔ19/Δ19 mice under a 12-h light/dark cycle. N = 4 for WT mice, N = 4 for ClockΔ19/Δ19 mice at each point. Statistical analyses were performed using a two-way ANOVA and Bonferroni’s test in order to compare differences of absolute mRNA level between WT and ClockΔ19/Δ19 mice at each time point. # P < 0.05, ## P < 0.01. (TIF) [file pone.0168234.s002.tif]

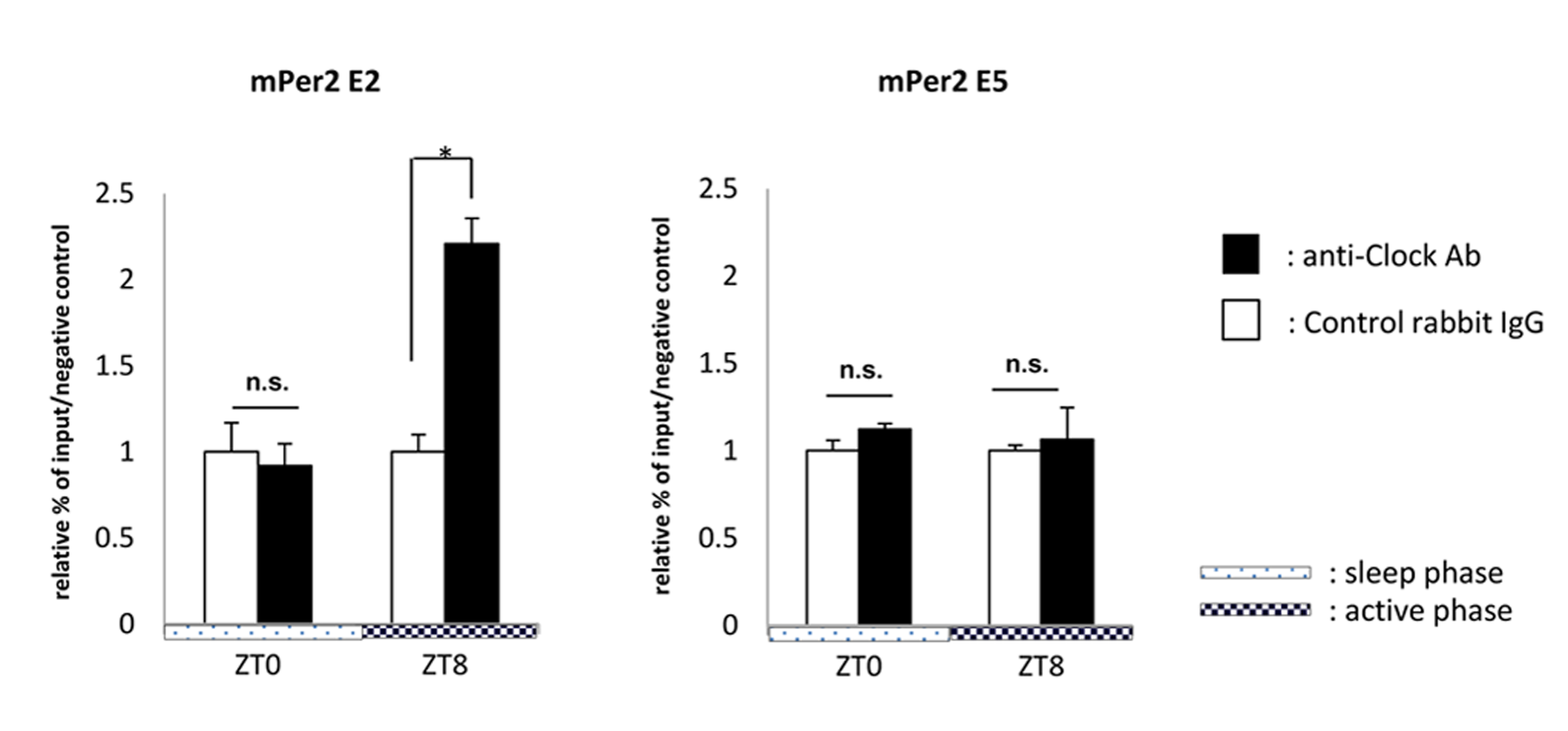

Supplement: S3 Fig — Each graph was described with the normalized value by the input DNA in each time point. Data are presented as a relative value of that of the control at each time point, and as means ± SE. (n = 3 for the Control group and anti-Clock Ab group at each time point). *P < 0.05, Mann-Whitney’s U-test, n.s., not significant. (TIF) [file pone.0168234.s003.tif]
